# Determinants in adolescence for adult sickness absence in women and men: a 26-year follow-up of a prospective population based cohort (Northern Swedish cohort)

**DOI:** 10.1186/1471-2458-13-75

**Published:** 2013-01-26

**Authors:** Ellenor Mittendorfer-Rutz, Gunnel Hensing, Hugo Westerlund, Magnus Backheden, Anne Hammarström

**Affiliations:** 1 Department of Clinical Neuroscience, Division of Insurance Medicine, Karolinska Institutet, Stockholm, Sweden; 2 Department of Public Health and Community Medicine, Institute of Medicine, University of Gothenburg, Social Medicine, The Sahlgrenska Academy, Gothenburg, Sweden; 3 Stress Research Institute, Stockholm University, Stockholm, Sweden; 4 Department of Learning, Informatics, Management and Ethics, Karolinska Institutet, Stockholm, Sweden; 5 Department of Public Health and Clinical Medicine, Umeå University, Umeå, Sweden

**Keywords:** Sickness absence, Gender, Socio-economic, Parental

## Abstract

**Background:**

To date little is known regarding how factors measured in adolescence predict sickness absence in adulthood, and whether different patterns of factors exist for women and men that could contribute to an explanation of adult gender differences in sickness absence.

**Methods:**

All pupils in the last year of compulsory school in the municipality of Luleå with complete information from surveys (questionnaires) in 1981 and 1983 (compulsory and upper-secondary schooling; 16 and 18 years of age, N=719) were followed with register data on medically certified sickness absence (1993–2007). Generalised linear models were applied to calculate Risk Ratios with 95% Confidence Intervals (CI) comparing annual mean numbers of sickness absence spells in exposed versus unexposed groups.

**Results:**

In the multivariate model, the following factors were found to be predictive of future sickness absence in women: participating in an upper secondary school program in 1983 dominated by women (> 60%): 1.41 (95% CI 1.00 – 1.97); sometimes sickness absence from school in 1981: 1.60 (95% CI 1.18 – 2.17) and low parental socioeconomic status in 1981: 2.20 (95% CI 1.44 – 3.38). In men, low school grades in 1981: 4.36 (95% CI 2.06 – 9.22) and fathers not in gainful employment in 1981: 2.36 (95% CI 1.53 – 3.66) were predictive.

**Conclusion:**

The findings suggest that sickness absence in adulthood is predicted by factors measured in adolescence. These predictors may differ for women and men. For women, early life absence and social environmental factors, for men low achievements at school and lack of employment of their father seem to be predictive.

## Background

### Gender differences in sickness absence

Women have higher sickness absence rates than men in most countries. The size of the gender gap is associated with structural relations of women and men on the labour market, such as the work force participation among women [1]. Sickness absence is clearly related to work and work environment as well as factors outside gainful work, like conflicts between paid and unpaid work and family responsibilities [1,2]. The effect of these factors on sickness absence varies between women and men, but studies taking variation of these factors into account could not entirely explain gender differences in adult sickness absence [1,3-6].

Still, gender differences in sickness absence patterns could also arise from factors measured before the entrance into the labour force, such as conditions during childhood and adolescents. Such conditions could influence the individual’s health directly, but also the selection into education and adult occupations which are important determinants of adult health and sickness absence. Only a few studies have investigated the impact of conditions during childhood and adolescence on sickness absence in adulthood [7-10]. The focus of these studies has been on factors related to individual health and behaviour as well as parental health and socio-economic status during childhood and adolescence. Here, gender differences in the importance of parental educational level were found, showing that parental low educational level had a stronger impact on men's than on women's risk for sickness absence in adulthood [7].

Few studies, however, have included the school environment. Recent longitudinal studies have shown that the psychosocial environment of the school class was associated with adult health [11]. One characteristic of school classes at the high school level is that they often are gender segregated. A possible impact of gender-segregated schooling on the future propensity to take sickness absence has to our knowledge not been studied to date. British sociological work has shown how courses in caring, that are typically female dominated educations, also involve a development of a gendered and classed subjectivity related to moral virtue and domestic duties [12]. Probably, male dominated courses have similar processes but with another content. Hansen et al. found that men with traditional male role norms had lower levels of sickness absence than men with less traditional norms [13].

Recent evidence suggests that women in women-dominated work-places have more sickness absence [14], which is consistent with the perception of gender being a considerable factor in sickness absence cultures [15]. Studies have shown that occupational values are developed during adolescence and that this development can be different for women and men [16,17]. Thus, it is possible that absence cultures already develop in adolescence. We hypothesised that participation in a female-dominated educational program may be associated with future higher sickness absence levels. The pathway could be early identification with a gendered subjectivity involving moral virtues and domestic duties that might promote these women’s domestic attachment and reduce their attachment to paid work. A culture of gendered subjectivity in women dominated educations supportive of being sickness absent rather than being sickness present, could be reinforced also in female dominated paid work. In line with this hypothesis we expect women who did not participate in female dominated programs and men to have lower future levels of sickness absence.

### Life course paradigm

The limited scientific interest relating conditions during childhood and adolescence to adult sickness absence is surprising, given that childhood adversities have repeatedly been associated with adult health and function [18-20] and that a life-course perspective is a much advocated approach in epidemiological research [18]. A life-course approach is based on the assumption that the impact of biological and social risk factors as well as protective factors may vary with age at exposure due to a differential impact at different developmental stages [18]. Any variations in risk for adverse health and functioning in adulthood may be due to the accumulation of exposures during the life span, due to a latency in outcome manifestation, or due to specific developmental mechanisms that act during a specific age window [18]. It can be argued that upper secondary school, where young people are for the first time split into different educational programmes based on their own vocation-oriented choices, often in a highly gendered way, may be a time window of special importance for the socialisation into gendered occupational roles which could entail different attitudes and behaviours related to health and work absenteeism.

### Aim

The aim of this longitudinal study was to examine parental, school and health related factors in adolescence as predictors of sickness absence in adulthood, and to find out whether different patterns emerged for women and men that could contribute to an explanation of adult gender differences in sickness absence.

## Methods

### Design

Data were drawn from a prospective, population-based cohort study in Northern Sweden (the Northern Swedish Cohort). The population consisted of all pupils in the last year of compulsory school in the municipality of Luleå in 1981 (n=1083, 577 boys, 506 girls). The cohort has been followed from 16 years of age in spring 1981–2007 (42 years old, 26-year of follow-up). The attrition rate was very low: up until 2007 information about 1010 persons (482 women and 519 men) was available, which constitutes 94% of the survivors (12 participants died). As the gender distribution in the educational programme during upper-secondary schooling was one of the analysed predictors, 223 participants were excluded because they did not proceed to upper-secondary schooling.

Due to missing information on three continuous variables, 59 participants were additionally excluded, resulting in 719 participants (339 women and 380 men). Sensitivity analyses revealed the comparability of estimates in the dataset including study participants advancing to upper-secondary schooling (N= 778) and the dataset subtracting the missing data on continuous variables (N=719).

### Outcome measures

Information on number of medically certified sickness absence spells per year from January 1993 to December 2007 was obtained from Statistics Sweden. The outcome was measured as annual number of sickness absence spells with economic compensation from the National Insurance Office. The Swedish sickness insurance covers all people living in Sweden, who are 16 years of age or older, and have at least a minimum annual income from work [21]. After the first day of sick leave (which is a qualifying day without compensation), sick pay is provided by the employer for the first 14 days of the period of absence, after which the employee can receive sickness benefit from the national social insurance system [21]. Register data is therefore not available for the first 14 days when the employer is responsible*.* The preconditions for the qualifying day and the time period including economic compensation by the employer varies for self-employed, unemployed and people with some chronic diseases.

### Exposure

Information on exposure was drawn from two surveys (questionnaires) carried out in 1981 and 1983 when the study participants were 16 and 18 years of age, respectively. All but three exposure variables were introduced as categorical variables, hereby coding missing values as categories.

Gender-segregated schooling during adolescence was operationalised as participation in a secondary school program with a high percentage of girls or boys, respectively. Information about participation in educational programmes during upper-secondary schooling (1983, 18 years of age) was obtained for each participant from lists over all pupils in different programmes from the high schools in the municipality of Luleå. For those who had moved to other municipalities, similar information was obtained from their respective schools. The programmes were categorised following the distribution of the proportion of participating women in the respective program (Low: < 30% women; Medium: 30-60% women; High: > 60% women). Grades in the elementary school certificate (1981) were obtained from the local municipality for each participant and were categorised from a continuous variable in four categories based on quartiles.

We classified frequency of any days of self-reported sickness absence (1981 and 1983) and truancy, measured in 1981 as follows: Often: "once per week" and " several times per month"; Sometimes: "once per month" and "several times during the term"; and no: "almost never". The question on sickness absence and truancy were formulated as follows: How often does it happen that you are away from school because you are sick? and How often does it happen that you are away from school without being sick? There were high correlations between these self-reported data and information about truancy (Pearson’s correlation 0.46 with p> 0.01) and sickness absence (Pearson’s correlation 0.27 with p> 0.01) from personal interviews about each pupil with their formal teachers at age 16. Information on the use of acid reflux medication was introduced as a dichotomous variable.

From the questionnaire, we calculated an additive sum of severe symptoms during the preceding 12 months (1981) and the number of health care visits to a physician during the preceding 12 months (1981). Information about the number of health care visits to the school nurse during the preceding 12 months (1981) was obtained from interviews with all school nurses responsible for grade nine pupils in Luleå in 1981. These three variables were introduced as continuous variables in the analyses. A six-item version of the General Health Questionnaire was used to assess psychological distress [22]. The variable was dichotomised as no symptoms and one symptom compared to more than one symptom.

Questionnaire information on maternal and paternal socio-economic group was based on type of occupation and categorised in three groups according to guidelines from Statistics Sweden [23]. We used either maternal or paternal socio-economic group, whichever was higher, in accordance with the dominance principle [24]. The dominance approach allows the labour market position of either parent (mother or father) to determine the social class of the family. This is in contrast to the conventional approach, which determines the social class position of the family by the occupation of the father. The dominance approach has been shown to perform well in classifying families' social class [24]. Parental employment status was introduced as a categorical variable, comparing employed to not employed (including unemployed, old-age- or disability pension or other). Only paternal employment status was used in the final model as maternal employment status was not associated with the outcome. Information on parental health status was based on two questions in the questionnaire (one about the mother and one about the father). Pupils answered to the following question: Is your mother/father healthy. The answer alternatives were: yes, as far as I know; no, somatic disorder; no, mental disorder; no, alcohol problems and I don’t know. The variables on the health status of both mother and father were dichotomised.

### Statistics

The number of sickness absent spells was analysed as counts using a Generalised linear model (Generalised Estimating Equation) for longitudinal design and assuming a negative binominal distribution. Analyses were carried out separately for women and men. Due to our interest in persons entitled to sick-leave benefits, individuals who were out of the labour force during a whole year, were judged as being not at risk for sickness absence and therefore the number of sickness absent spells was set to "missing" for the respective year. Mean numbers of sickness absence spells per year (including a 95% Confidence Interval, CI) over the total time period from 1993 to 2007 were estimated in univariate models. Crude and adjusted Risk ratios (RR) with 95% CI were calculated comparing annual mean numbers of sickness absence spells in the exposed groups with the reference groups, by including time (1993–2007) as a covariate in all models.

### Ethical considerations

The study has been approved by the Regional Ethics Vetting Board in Umeå.

## Results

An overview of the number and frequencies of all categorised explanatory variables is given in Table 1. Considerable differences between women and men emerged with regard to the proportions in different variables. Women participated more often than men in an educational programme consisting primarily of students of the same sex (79 vs. 64%) and women were twice as likely to obtain high school grades (42 vs. 21%). Women were also more likely than men to report more than one symptom in the General Health Questionnaire- 6 (58 vs. 44%). The other variables did not show any considerable differences in the proportions between women and men.

**Table 1:** Number, frequencies and mean number of sickness absence spells per year and 95% confidence intervals (CI)

	**Women**			**Men**		
**Characteristics**	**N**	**%**	**Mean N spell/year (95% CI)**	**N**	**%**	**Mean N spell/year (95% CI)**
**Proportion of women in educational program (18 yrs)**						
Low	12	4	0.15 (0.08 – 0.29)	244	64	0.13 (0.11 – 0.16)
Medium	59	17	0.14 (0.11 – 0.19)	82	22	0.06 (0.03 – 0.09)
High	268	79	0.21 (0.18 – 0.25)	54	14	0.11 (0.07 – 0.19)
**Truancy (16 yrs)**						
Never	167	49	0.17 (0.14 – 0.21)	162	43	0.10 (0.08 – 0.13)
Sometimes	137	41	0.19 (0.16 – 0.23)	160	42	0.11 (0.08 – 0.14)
Often	34	10	0.36 (0.21 – 0.62)	55	15	0.15 (0.10 – 0.22)
**School grades (16 years)**						
Low	9	3	0.28 (0.18 – 0.43)	61	17	0.21 (0.16 – 0.29)
Medium	171	50	0.20 (0.17 – 0.24)	201	54	0.10 (0.08 – 0.12)
High	142	42	0.18 (0.13 – 0.24)	79	21	0.04 (0.03 – 0.07)
**Individual health (16 yrs)**						
*Sickness absence*						
Never	105	31	0.13 (0.10 – 0.16)	134	35	0.12 (0.09 – 0.16)
Sometimes	216	64	0.23 (0.19 – 0.28)	226	60	0.10 (0.08 – 0.13)
Often	18	5	0.20 (0.13 – 0.30)	18	5	0.17 (0.08 – 0.36)
**Individual health (18 yrs)**						
*GHQ 6*						
> 1	197	58	0.21 (0.17 – 0.26)	169	44	0.11 (0.09 – 0.15)
0/1	138	41	0.17 (0.14 – 0.21)	199	52	0.11 (0.08 – 014)
*Sickness absence*						
Never	154	45	0.16 (0.13 – 0.19)	185	49	0.10 (0.07 – 0.13)
Sometimes	174	51	0.23 (0.19 – 0.29)	177	47	0.13 (0.10 – 0.16)
Often	11	4	0.25 (0.17 – 0.39)	18	4	0.10 (0.06 – 0.19)
**Parental socio–economic status and health (16 yrs)**						
*Parental SES*						
Low	31	9	0.23 (0.19 – 0.28)	40	11	0.14 (0.11 – 0.18)
Middle	203	60	0.20 (0.16 – 0.24)	199	52	0.09 (0.07 – 0.12)
High	103	30	0.09 (0.06 – 0.12)	130	34	0.12 (0.07 – 0.22)
*Father's employment status*						
Employed	304	90	0.19 (0.17 – 0.23)	341	90	0.10 (0.08 – 0.12)
Unemployed	6	2	0.30 (0.16 – 0.56)	3	1	0.23 (0.10 – 0.54)
Old – age/disability pension	7	2	0.21 (0.11 – 0.41)	11	3	0.24 (0.14 – 0.40)
Other	22	6	0.22 (0.16 – 0.30)	25	7	0.18 (0.11 – 0.30)
*Father's health status*						
Healthy	262	77	0.19 (0.16 – 0.23)	306	81	0.11 (0.09 – 0.14)
Not healthy	71	21	0.22 (0.18 – 0.26)	63	16	0.11 (0.08 – 0.16)
*Mother's health status*						
Healthy	274	81	0.20 (0.16 – 0.23)	318	84	0.11 (0.09 – 0.14)
Not health	62	18	0.21 (0.16 – 0.27)	58	15	0.10 (0.07 – 0.16)

Percentage does not always add up to 100% due to missing values, all results derived from univariate models.

Table 1 additionally indicates the mean number of sickness absence spells per year and 95% Confidence intervals (CI) derived from univariate models. Women had in general higher mean numbers of sickness absence spells per year in all categories of all variables. Mean annual number of spells ranged from 0.04 (high grades in school) among men to 0.36 (often truant at school) among women. In the following categories the mean annual number of spells in women was more than twice the mean number in men: medium proportion of women in the educational program; often truant from school; high grades in school certificates; sometimes and often sickness absent at 16 and 18 years of age, respectively, medium parental socio-economic group and mothers not healthy

### Risk factors for future sickness absence in women

In Table 2 crude and multivariate adjusted Risk Ratios for future sickness absence among women are presented for the various characteristics. Participating in an educational program with a high proportion of women was associated with a 49% increased risk of future sickness absence compared to participation in a program with a medium proportion of women. The risk decreased somewhat but the association remained significant in the multivariate model (41% risk increase). At 16 years of age reporting being sometimes sickness absent from school compared to never, and having parents with low and medium socio-economic status compared to a high status remained significant predictors in the multivariate model. While adolescent sickness absence increased the risk of adult sickness absence with 60%, women reporting the parental socio-economic status in adolescence as low had a 2.2 fold risk for adult sickness absence.

**Table 2:** Crude and adjusted Risk ratios (RR) of mean number of sickness absence spells per year, 339 women

**Characteristics**	**Model I**	**Model II**
	**RR (95 % CI)**	**RR (95 % CI)**
**Proportion of women in educational program (18 yrs)**		
Low	1.03 (0.50 – 2.15)	1.04 (0.51 – 2.14)
High	1.49 (1.07 – 2.09)	1.41 (1.00 – 1.97)
Medium	1	1
**Truancy (16 yrs)**		
Often	2.09 (1.17 – 3.72)	1.74 (0.84 – 3.58)
Sometimes	1.12 (0.87 – 1.43)	0.91 (0.68 – 1.21)
Never	1	1
Missing	1.05 (0.88 – 1.26)	1.51 (0.92 – 2.74)
**School grades (16 yrs)**		
Low	1.56 (0.92 – 2.63)	1.03 (0.51 – 2.07)
Medium	1.11 (0.79 – 1.57)	0.95 (0.65 – 1.38)
High	1	1
Missing	1.56 (0.92 – 2.63)	1.03 (0.51 – 2.07)
**Individual health (16 yrs)**		
*Sickness absence*		
Often	1.55 (0.97 – 2.49)	1.12 (0.65 – 1.93)
Sometimes	1.81 (1.33 – 2.47)	1.60 (1.18 – 2.17)
Never	1	1
*Nr. nurse visits*		
Change in risk per visit	0.96 (0.88 – 1.04)	0.94 (0.87 – 1.01)
*Nr. physician visits*		
Change in risk per visit	1.05 (0.99 – 1.11)	1.04 (0.98 – 1.12)
*Sum of symptoms*		
Increased risk per symptom	1.07 (1.01 – 1.13)	0.99 (0.93 – 1.05)
**Individual health (18 yrs)**		
*GHQ 6*		
> 1	1.23 (0.93 – 1.62)	1.11 (0.86 – 1.42)
0/1	1	1
Missing	1.58 (1.09 – 2.30)	0.75 (0.38 – 1.50)
*Sickness absence*		
Often	1.64 (1.04 – 2.60)	1.44 (0.85 – 2.45)
Sometimes	1.49 (1.13 – 1.97)	1.34 (0.99 – 1.81)
Never	1	1
**Parental socio – economic status and health (16 yrs)**		
*Parental SES*		
Low	2.62 (1.75 – 3.92)	2.20 (1.44 – 3.38)
Middle	2.24 (1.49 – 3.38)	1.95 (1.31 – 2.91)
High	1	1
Missing	3.88 (1.93 – 7.77)	4.18 (1.49 – 11.71)
*Father's employment status*		
Not employed*	1.16 (0.87 – 1.55)	1.06 (0.76 – 1.48)
Employed	1	1
*Father's health status*		
Not healthy	1.12 (0.85 – 1.47)	1.10 (0.83 – 1.44)
Healthy	1	1
Missing	1.17 (0.68 – 2.04)	1.01 (0.52 – 1.96)
*Mother's health status*		
Not healthy	1.07 (0.80 – 1.45)	0.88 (0.63 – 1.24)
Healthy	1	1
Missing	1.00 (0.41 – 2.44)	0.81 (0.30 – 2.19)

Model I: All variables are adjusted for time. Model II: all variables are adjusted for each other and time. * not employed: unemployed, old–age– and disability pension and other.

Being often truant from school compared to never, and reporting sickness absence from school at 18 years of age, were associated with an increased risk only in the univariate analyses. Also the association of an increasing number of self-reported symptoms with sickness absence in adulthood did not remain significant in the multivariate model. The remaining factors were not associated with an increased risk of sickness absence in women.

### Risk factors of future sickness absence in men

Among men two factors reported at 16 years of age remained significant predictors for sickness absence in adulthood in the multivariate model, namely low and medium school grades compared to high grades, and reporting the father not being employed compared to employed (Table 3). Low school grades and having a father without employment increased the risk of future sickness absence 4.3 and 2.3 times, respectively. Participating in an educational program with low and high proportions of women compared to a medium proportion increased the risk of sickness absence in adulthood in men only in the univariate model. None of the other health and behaviour related factors were significantly associated with future sickness absence among men.

**Table 3:** Crude and adjusted Risk ratios (RR) of mean number of sickness absence spells per year, 380 men

**Characteristics**	**Model I RR**	**Model II RR**
	**(95 % CI)**	**(95 % CI)**
**Proportion of women in educational program (18 yrs)**		
Low	2.35 (1.42 – 3.90)	1.42 (0.78 – 2.60)
High	2.02 (1.00 – 4.08)	1.58 (0.78 – 3.20)
Medium	1	1
**Truancy (16 yrs)**		
Often	1.49 (0.92 – 2.42)	0.86 (0.50 – 1.46)
Sometimes	1.05 (0.72 – 1.53)	0.86 (0.59 – 1.25)
Never	1	1
Missing	4.19 (1.02 –17.23)	3.90 (0.84 – 18.04)
**School certificate (16 years)**		
Low	4.80 (2.59 – 8.91)	4.36 (2.06 – 9.22)
Medium	2.28 (1.28 – 4.07)	2.24 (1.25 – 4.02)
High	1	1
Missing	4.81 (2.59 – 8.91)	4.36 (2.06 – 9.22)
**Individual health (16 yrs)**		
*Sickness absence*		
Often	0.85 (0.59 – 1.21)	1.30 (0.57 – 3.00)
Sometimes	1.44 (0.65 – 3.20)	0.79 (0.54 – 1.15)
Never	1	1
Missing	1.75 (1.31 – 2.33)	0.16 (0.03 – 0.88)
*Nr. nurse visits*		
Change in risk per visit	0.92 (0.80 – 1.06)	0.98 (0.86 – 1.11)
*Nr. physician visits*		
Change in risk per visit	0.92 (0.80 – 1.06)	0.96 (0.84 – 1.08)
*Sum of symptoms*		
Increased risk per symptom	0.98 (0.86 – 1.12)	1.02 (0.89 – 1.17)
*Use of anti–acid medication*		
*Yes*	1.70 (0.99 – 2.90)	1.25 (0.72 – 2.18)
*No*	1	1
**Individual health (18 yrs)**		
*GHQ 6*		
*> 1*	1.11 (0.78 – 1.57)	0.97 (0.68 – 1.38)
*0/1*	1	1
Missing	0.94 (0.42 – 2.09)	0.57 (0.25 – 1.31)
*Sickness absence*		
Often	1.08 (0.56 – 2.10)	0.64 (0.31 – 1.31)
Sometimes	1.34 (0.94 – 1.91)	1.29 (0.89 – 1.88)
Never	1	1
**Parental socio–economic status and health (16 yrs)**		
*Parental SES*		
Low	1.19 (0.62 – 2.30)	0.95 (0.48 – 1.87)
Medium	0.73 (0.38 – 1.42)	0.75 (0.39 – 1.44)
High	1	1
Missing	0.96 (0.37 – 2.54)	0.84 (0.29 – 2.24)
*Father's employment status*		
Not employed*	2.01 (1.35 – 2.98)	2.36 (1.53 – 3.66)
Employed	1	1
*Father's health status*		
Not healthy	0.97 (0.65 – 1.45)	0.72 (0.47 – 1.10)
Healthy	1	1
Missing	0.66 (0.39 – 1.12)	0.31 (0.15 – 0.66)
*Mother's health status*		
Not healthy	0.91 (0.57 – 1.43)	0.91 (0.57 – 1.46)
Healthy	1	1
Missing	0.40 (0.14 – 1.11)	0.39 (0.10 – 1.46)

Model I: All variables are adjusted for time. Model II: all variables are adjusted for each other and time. * not employed: unemployed, old–age– and disability pension and other.

## Discussion

In this 26-year prospective analysis of school leavers, it was found that participating in an upper secondary school program dominated by women, sickness absence from school and lower adolescent socio-economic status, was predictive of future sickness absence in the fully adjusted model in women, while lower school grades and having fathers not in gainful employment in childhood were predictive in men.

### Methodology

The major strength of this study is the prospective analysis of a community-based cohort of school leavers with exceptionally low attrition followed over almost three decades with repeated measurements and register data. Earlier research has shown that the non-responders tend to have worse health status – and thus increased risk of sick-leave - than the responders [25]. Another strength was the use of highly reliable public register data to measure sick-leave for each individual, which minimises bias and loss to follow-up. This cohort of school leavers from a town in Northern Sweden has been shown to be representative of the whole country in relation to socio-economic background factors and health in adolescence, [26,27]. The present cohort therefore consists of pupils, who have both accomplished the 9 years of compulsory schooling and who proceeded to an additional three-years of upper-secondary schooling. The results of this study may therefore be generalisable to a generation of Swedish school leavers of upper secondary school.

Using register data on sickness absence as the outcome measure entails advantages compared to self-reported sickness absence with regard to the validity of data [28,29]. Register data on absences also implies that spells are of a certain minimum length, meaning that some of those classified as having no absences could in fact have had frequent, short-term absences, which might have led to an underestimation of these absences. Another consequence is that we may be unable to elucidate one of the most pronounced differences in sickness absence between women and men, which is in shorter spells. Using mean number of spells as the outcome variable rather than total sickness absence days, on the other hand, may mean that the influence of severe health problems is underestimated, as such problems are likely to result in many and/or long absence spells. While it is unique in this research field to have access to data on sickness absence during adolescence, we had no information on the number of days of self-reported sickness absence in adolescence. Future studies should consider measures on length of sickness absence in adolescence when investigating the association of absence from school and adult sickness absence.

Another limitation is the size of the population, with risk for power problems for some specific variables and type-2 errors. A further limitation is that possible interactions between the various predictors and time could not be analysed in the models as the algorithm became unstable. In addition, the validity of the questionnaire data at age 16 and 18 on truancy and sickness absence needs to be discussed. Both school absence due to sickness or due to truancy can be interpreted as a lack of “regular social participation” and might be difficult to clearly discriminate. A common understanding would be that the pupils would underestimate especially their truancy. Still, a strong correlation between questionnaire data and teachers’ interviews on truancy was found which suggests that truancy had good validity.

### Gender-segregated schooling

Participating in an upper secondary school program dominated by women was significantly associated with adult sickness absence in the fully adjusted model in women. Two main explanations can be suggested. First, there might be a more indirect way related to gender construction and gendered subjectivity as mentioned in the introduction. Secondly, the association can be explained by selection mechanisms since female-dominated educations tend to lead to women-dominated occupations which have been shown to have lower salaries and status, less control, and work tasks which differ systematically from those of most male occupations [30]. Still, Laaksonen found that occupation and workplace explained only about one third of the difference in sickness absence between women and men [6]. In the present study, we did not adjust for occupation and work environment in adulthood, as this could have resulted in over-adjustment. An alternative explanation is that girls choosing female-dominated education programs could be negatively selected in terms of health or occupational motivation. However, adjustment for individual health at ages 16 and 18, school grades, and truancy did not attenuate the relationship between female domination and sickness absence, which suggests that such selection mechanisms are unlikely.

### Socio-economic status

Low parental socio-economic status and lack of paternal employment measured during adolescence were associated with increased risk of sickness absence in adulthood in women and men, respectively. These findings are in line with previous studies on the impact of childhood socio-economic adversities on sickness absence [7] and disability pension [31]. Kristensen et al. reported a higher population attributable risk for sickness absence due to musculoskeletal diagnoses related to low parental educational level for boys than for girls [7]. Future studies are warranted in order to further elucidate potential gendered patterns in the effect of low parental socioeconomic status for offspring's risk of sickness absence.

### Tracking of sickness absence

Girls reported somewhat higher levels of sickness absence than boys already at ages 16 and 18, and in women, high sickness absence in adulthood was predicted by sometimes, as opposed to never, being sickness absent at age 16. This association remained significant after adjustment for other health variables. There was also an association between sickness absence at age 18 and adult sickness absence, which, however, did not remain after adjustments. These findings might give an indication that sickness absence, largely unrelated to health, could be established already before entering into upper-secondary education, at least among women, and be associated with sickness absence in adulthood. However, these findings should be interpreted with caution as these associations were only found among women and only for reporting being sickness absent sometimes. The lack of significant findings for men and for often sickness absence for women could possibly be explained by lower power. Previous studies found either a weak or no association between absence from school in childhood and adolescents, and sickness absence in adulthood [9,10,32]. Two of these studies could, however, not distinguish between absence due to sickness and truancy. In the present study, self-reported truancy in adolescence was found to be a significant predictor for adult sickness absence in women, only in the univariate analysis. Future studies are warranted to introduce even more detailed questions on sickness absence and truancy in studies on adolescent absence from school and adult sickness absence, in order to better discriminate sickness absence from truancy.

### School grades

In men, school grades at age 16 were related to sickness absence in adulthood, while such an association was lacking in women. We have found no studies specifically linking school grades to adult sickness absence. There are several plausible links, such as intelligence, personality, social class, chronic diseases, health behaviours, and educational achievement, and an established pattern of high absenteeism could have resulted in lower academic achievement already at age 16. The much weaker association in women than in men is more difficult to explain. Earlier studies did not report considerable gender differences in the association of school grades with subsequent health and health behaviour outcomes [33-35]. Future studies are warranted both for replication of these findings and in order to scrutinise potential underlying mechanisms.

## Conclusion

The results of this study indicate that women-dominated education in upper secondary school as well as tracking of absenteeism patterns established in school may have contributed to the higher rates of sickness absence in adult women, but further research is needed to rule out alternative explanations for the findings. Socioeconomic circumstances in adolescence were also predictive of adult sickness absence, with different indicators being predictive in women and men, which warrants further exploration. Low school grades were the strongest predictors in men.

## Competing interests

The authors declare that they have no competing interests.

## Authors’ contribution

The study was jointly designed by all authors. MB and EMR were responsible for the data analyses. AH was the PI of the cohort study and main responsible for data acquisition and ethical approvals. Findings were jointly interpreted by all authors. EMR drafted the paper outline. All authors contributed to successive drafts. The final manuscript was approved by all authors.

## Pre-publication history

The pre-publication history for this paper can be accessed here:

http://www.biomedcentral.com/1471-2458/13/75/prepub
